# Comprehensive Methodology for Evaluating the Drug Loading of Iron Oxide Nanoparticles Using Combined Magnetometry and Mössbauer Spectroscopy

**DOI:** 10.3390/molecules30030676

**Published:** 2025-02-04

**Authors:** Nicusor Iacob, Petru Palade, Cezar Comanescu, Ovidiu Crisan, Luiza Izabela Toderascu, Gabriel Socol, Gabriel Schinteie, Victor Kuncser

**Affiliations:** 1 National Institute of Materials Physics, 077125 Magurele, Romania; nicusor.iacob@infim.ro (N.I.); palade@infim.ro (P.P.); cezar.comanescu@infim.ro (C.C.); ocrisan@yahoo.com (O.C.); 2 Faculty of Physics, University of Bucharest, 077125 Magurele, Romania; 3 National Institute for Laser, Plasma and Radiation Physics, 077125 Magurele, Romania; izabela.jinga@inflpr.ro (L.I.T.); gabriel.socol@inflpr.ro (G.S.); 4 Faculty of Chemistry, University of Bucharest, 050663 Bucharest, Romania

**Keywords:** iron oxide nanoparticles, doxorubicin, surface molecule loading, magnetometry, Mössbauer spectroscopy, magnetic structure

## Abstract

A methodology for the quantitative estimation of the drug loading of iron oxide-based magnetic nanoparticles by corroborating magnetometry and Mössbauer spectroscopy investigations is reported. The proposed methodology is exemplified in the case of two series of nanoparticles, namely Fe_3_O_4_ nanoparticles covered with citric acid molecules and further functionalized with doxorubicin, and Fe_3_O_4_ nanoparticles covered with L-Cysteine molecules and further functionalized with doxorubicin. The general idea of the proposed methodology is to probe the real magnetic structure of the magnetic core via low-temperature Mössbauer spectroscopy for the correct estimation of the spontaneous magnetization of the magnetic core. It subsequently uses the ratio between the spontaneous magnetization of the covered nanoparticles and that of the magnetic core for the reliable and nondestructive evaluation of the nanoparticle loading by organic molecules. Although the methodology is exemplified in the case of magnetite-based nanoparticles, it can be successfully considered for a large class of medicine-loaded Fe-containing magnetic nanoparticles where ^57^Fe Mössbauer spectroscopy can be applied.

## 1. Introduction

A special role in biomedicine is played by magnetic nanoparticles (MNPs) with a wide range of functionalities, derived from their material nature, structure, and morphology; magnetic properties; and chemical affinity to biomolecules of medical interest [1,2]. A crucial condition that must be fulfilled in biomedical applications is the biocompatibility of the materials involved. The interaction between MNPs and biomolecules has opened a wide area of applications in biomedical fields, such as magnetic hyperthermia and drug delivery, used in cancer therapy [3,4], biomedical sensing [5] and imaging, etc. [6]. “Cancer” refers to the uncontrolled cellular growth in specific tissues that enhances angiogenesis and the formation of metastases [3,7]. Most cancers are associated with genetic alterations that affect the regulation of cellular growth. The treatment of cancer mainly focuses on stopping the chaotic cellular replication process through drug administration (cytostatic), which generally has a low degree of uptake by the malignant cells and aggressive side effects [8]. A novel way to solve this issue is to bind cytostatic molecules to MNPs, which can be directed by various procedures, including magnetic fields, to the target tumor. Following the uptake process in tumor cells where the MNPs are directed, i.e., by maintaining healthy cells almost unaffected [9,10,11,12,13], this treatment becomes more localized, efficiently releasing the drug molecules into the cells. Generally, many routes have been developed for targeting tumors through recent nanotechnology advances in the biomedical field. Drug carriers like polymeric nanoparticles, liposomes, viral and gold nanoparticles, carbon-based systems, and magnetic nanoparticles (e.g., iron oxides) were developed to optimize biomolecule transportation into cancer cells [14,15,16,17]. There are two targeting approaches for antitumor agents as follows [3,18,19,20]: (i) passive targeting, generated by the enhancement of permeability and the retention (EPR) effect, which allows the accumulation of high-molecular-weight molecules and fine particles (diameter ~20–500 nm) within the tumor tissue due to a leaky vasculature (notably, this targeting method has a universal role in treating different types of cancers using the same drug delivery system); and (ii) active targeting, involving the chemical bonding between membrane receptors from targeted cells and additional ligands bound on the surface of the drug carrier. Drug molecules used in cancer treatment are not easily bound to the surface of nanoparticles because of their lack of chemical affinity. Instead, an organic layer with a high affinity for the biomolecules of interest usually covers the surface of the nanoparticles. Depending on the type of cytostatic, specific organic layers must be used with respect to the biocompatibility conditions. Doxorubicin, a widely used drug in cancer treatment, has proven to be highly effective [21,22,23,24]. It treats various solid tumors, acting through multiple mechanisms [25]. It is important to note that depending on the dose, doxorubicin may induce cardiotoxicity, especially in a cumulative dose regime [26]. Therefore, the sensitive evaluation and control of drug loading is crucial for optimizing clinical treatment doses. There are specific methods for the evaluation of drug loading capacity such as analytical chemistry methods (high-performance liquid chromatography—HPLC [27,28]) or IR spectrophotometry techniques [29,30,31]. However, such methods focus more on the drug release capacity, which is not always straightforwardly related to the drug loading capacity. On the other hand, the advantage of using MNPs as nanocarriers of drug molecules is not only due to the additional targeting possibilities intermediated by specific configurations of applied magnetic fields but also due to the ability to finely control drug release through hyperthermia activation using low amplitude ac magnetic fields, which can superpose, in certain circumstances, with the localized curing effect introduced by the hyperthermia process itself. In this respect, the change in the magnetic properties of the core MNPs induced by the presence of medicine molecules might also become important.

This work presents an innovative methodology for quantifying the drug loading capacity of iron oxide-based MNPs by corroborating magnetometry and Mössbauer spectroscopy investigations. In a previous work [30], the doxorubicin cytostatic used in the treatment of melanoma was bound on the surface of magnetite-like MNPs through L-Cysteine molecules and tested in vitro on mouse 16F10 and human A375 metastatic melanoma cells. Following this, the proposed methodology is applied on two sets of iron oxide MNP systems, as follows: (i) Fe_3_O_4_ MNPs covered with Citric Acid (CA) and functionalized with doxorubicin and (ii) Fe_3_O_4_ MNPs covered with L-Cysteine (LC) and also functionalized with doxorubicin. Superconducting Quantum Interference Device (SQUID)-based Magnetometry and Mössbauer spectroscopy measurements of naked and successively loaded (by CA, CA&Dox, LC, and LC&Dox) Fe_3_O_4_ MNPs revealed the main information about the mass magnetization (in emu/gram) and magnetic structure on the nanoparticles’ surface, as influenced by the attached molecules. The evolution of saturation magnetization imposed by the non-magnetic loading is corroborated with the change in the magnetic state of the core of the MNPs in order to extract valuable information on their loading with medicine molecules.

The proposed methodology presents the following peculiarities and advances as compared to the existing ones exemplified above: (i) it provides direct information on the real loading capacity, rather than indirect, through the release capacity, (ii) it is not destructive, and (iii) it is really comprehensive, providing valuable information on the magnetic configuration of the core MNPs of importance with respect to the magnetic actuation capacity, as well as revealing the chemical bound mechanism of the organic molecules at the nanoparticle surface.

## 2. Results and Discussion

A complex structural and morphological characterization of all obtained samples has already been reported in [30,32] and therefore only the conclusion of interest for this work will be resumed here. In the case of naked Fe_3_O_4_ MNPs, similar broad size distributions ranging from 3 nm to 20 nm were evidenced in all preparations, with two types of morphologies as follows: spherical shape (for the finest particles) and faceted shape of a cubic or rhombohedral type (for the larger ones). Notably, a change in the morphological parameters (e.g., a slightly larger size distribution extending up to 30 nm) was observed only for the Fe_3_O_4_@LC@Dox MNPs, indicating the possible influence of the covering organic molecules on the magnetic configuration of the inorganic core only for this branch of samples.

**Figure 1:**
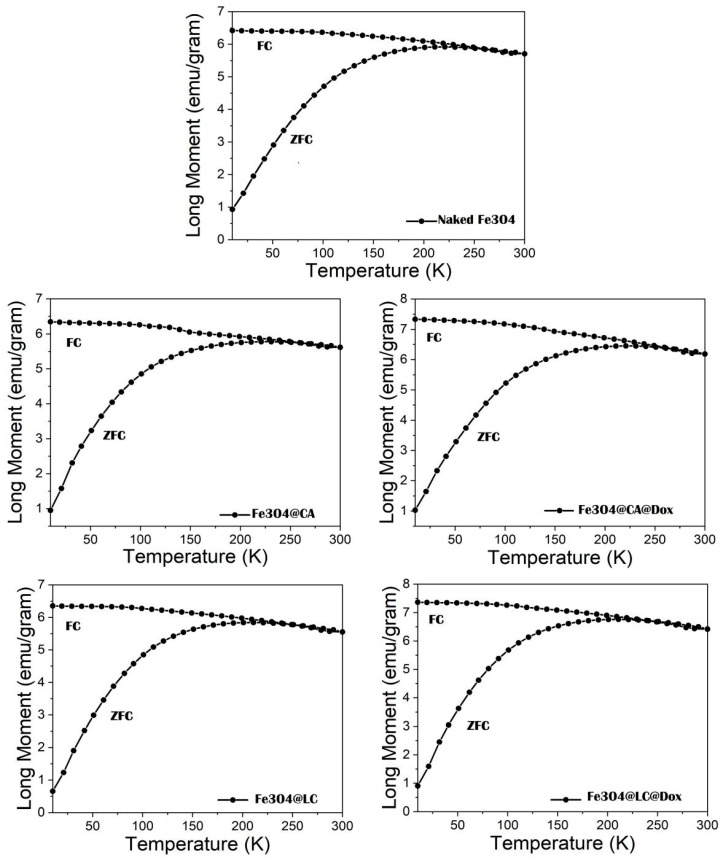
ZFC-FC curves measured in the temperature range from 10 K to 300 K in 100 Oe applied field on the investigated samples. Sample labeling is presented in the legend.

The ZFC–FC measurements from 10 K to 300 K in 100 Oe show a broad maximum on the ZFC curve in the range of 190–230 K, depending on the investigated samples (see Figure 1). So, blocking temperatures, T_B_ ranging from 210 to 222 K for different samples can be found from the maximum of the ZFC curves (see Table 1 and Table 2). Notably, T_B_ represents the temperature where the transition from the magnetically frozen regime to the superparamagnetic regime of MNPs takes place within the time windows of the measuring method (e.g., this is in the range from 1 to 100 s in the case of magnetic measurements). The flat shape of this maximum, along with the smooth divergence of the ZFC and FC curves, points to a wide distribution of the anisotropy energies (KV) of MNPs, where K is the anisotropy constant, and V is the volume of MNPs. However, the FC curve always develops over the ZFC curve, supporting the nature of magnetic monodomain nanoparticles. These results not only reveal the very broad size distribution of the naked and functionalized MNPs, especially extended at larger sizes (please see the findings confirmed by previous TEM investigations, according to [30]), but also quite similar magnetic relaxation phenomena that are specific to all investigated systems.

**Figure 2:**
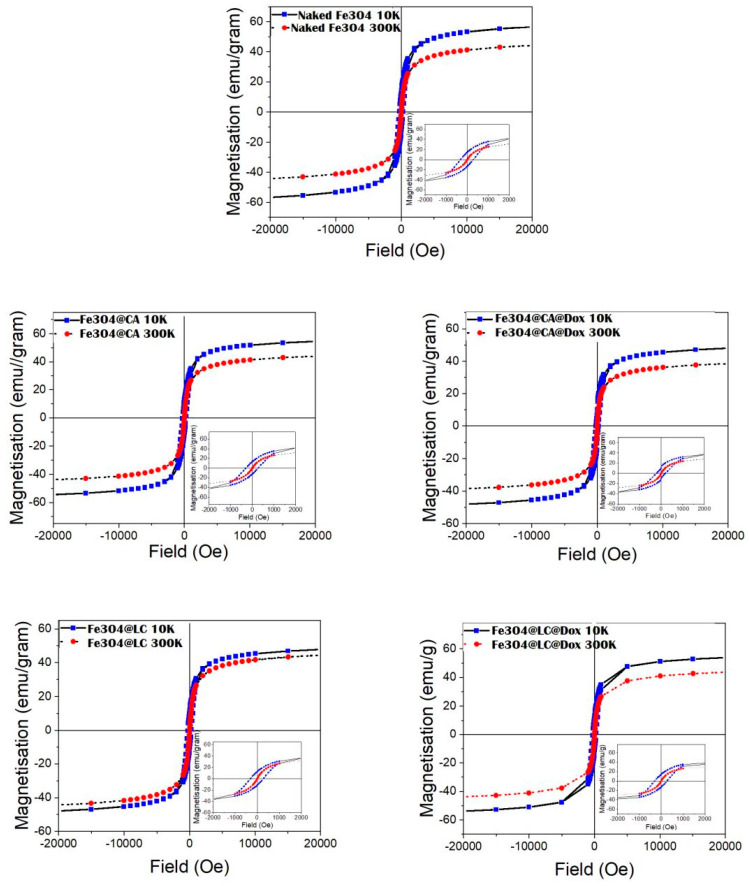
Magnetic hysteresis loops collected at 10 K (blue rectangle) and 300 K (red circle) in fields lower than 20,000 Oe for all considered samples. Sample labeling is presented in the legend. Insets present the hysteresis loops with an increased resolution in magnetic fields.

Due to the weak decrease in the magnetization curves between the branching point and 300 K, it can be assumed that only a fraction of the MNPs reached the superparamagnetic regime at 300 K, with the rest remaining in the frozen regime. According to the hysteresis loops presented in Figure 2, the coercive fields decrease from about 300 Oe at 10 K to about 15 Oe at 300 K for all samples. The very low value of the coercive field at 300 K, not specific to well-structured magnetite, represents additional proof that at this temperature, the system consists of a superposition of superparamagnetic and magnetically frozen MNPs. Magnetic hysteresis loops at 10 K show a saturation magnetization between 52 and 63 emu/g for all samples, values which are approximately 30–40% lower than the saturation magnetization of well-formed Fe_3_O_4_ nanoparticles [33]. Notably, the saturation magnetization obtained at the lowest temperature of 10 K (full magnetic frozen regime) provides the best estimation for the spontaneous magnetization, which can be directly connected with the real magnetic structure of MNPs. A law of approach [34] has been used for the sensitive evaluation of saturation magnetization, as indicative of the spontaneous magnetization values reported in Table 1 and Table 2.

The obtained low value of the saturation magnetization at 10 K indicates the formation of a quite defect magnetite phase or even the progressive formation of a maghemite phase at the particle surface (the maghemite is itself an intrinsically defect magnetite with cation vacancies [35]). Such a crystalline defect structure may lead to a magnetic defect structure at the particle surface, which, on one side, decreases the saturation magnetization and, on the other side, is sensitive to the surface molecules chemically bound to the nanoparticle (NP) surface. To provide complementary information on the magnetic structure of naked and functionalized MNPs, low-temperature Mӧssbauer spectra were recorded for all samples. Contrary to the magnetic data showing almost no difference between ZFC-FC and as well as between hysteresis loops for the naked Fe_3_O_4_ in the two series, slight differences between the magnetic relaxation phenomena within the Mössbauer time window (10^−9^ s) were observed, as subsequently discussed.

**Figure 3:**
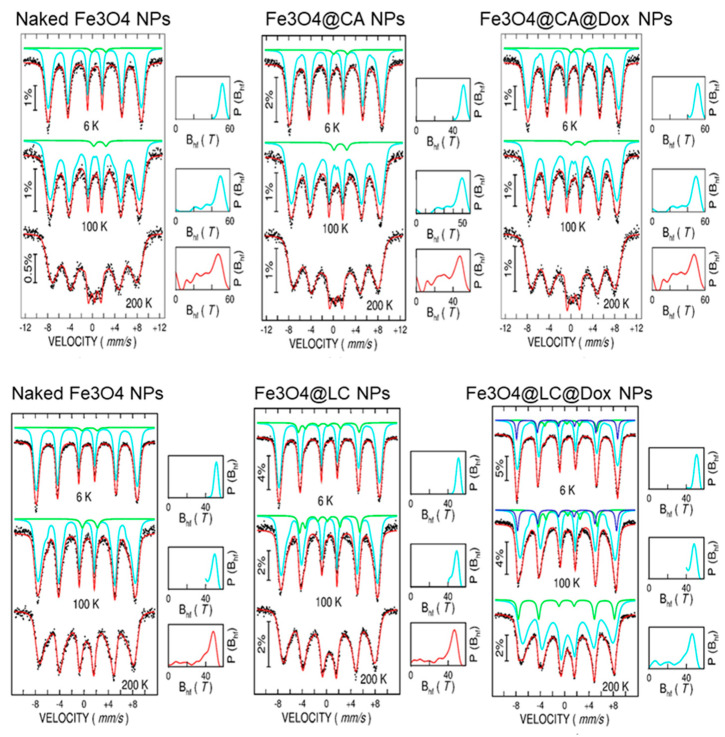
Temperature-dependent Mӧssbauer spectra were collected from naked and functionalized Fe_3_O_4_ NPs, according to the top labeling. The probability distribution of the magnetic hyperfine field is shown on the right hand of each spectrum.

The spectra collected at three temperatures (6 K, 100 K, and 200 K) for naked Fe_3_O_4_ and NPs loaded with L-Cysteine and L-Cysteine and doxorubicin (Fe_3_O_4_@LC, Fe_3_O_4_@LC @Dox), as well as for a second series of naked Fe_3_O_4_ NPs and NPs loaded with citric acid and citric acid and doxorubicin (Fe_3_O_4_@CA and Fe_3_O_4_@CA@Dox), are shown in Figure 3.

**Figure 4:**
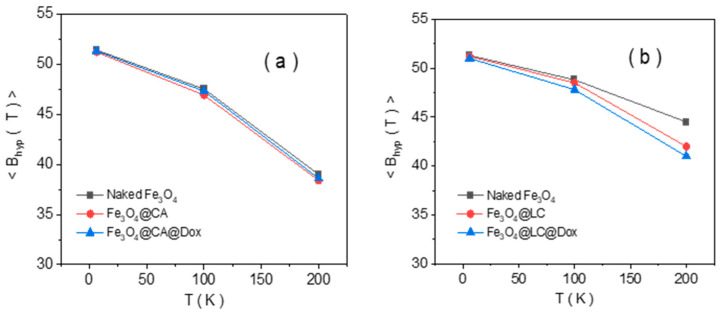
The evolution of the average magnetic hyperfine field versus temperature for naked and MNPs functionalized with CA (**a**) and for naked and MNPs functionalized with LC (**b**).

The Mӧssbauer spectra at the lowest temperature of 6 K consist, in all cases, of a broad sextet, as expected in the case of Fe oxide MNPs, with distributed configurations of Fe ions. The sextets become even broader at increasing temperatures, showing collapsing behavior that starts at 200 K and is specific to MNPs in the magnetic relaxation regime. This aspect leads to the possibility of having a unitary fitting of all spectra at increasing temperatures via a distribution of magnetic hyperfine fields, according to the algorithm proposed in [36]. The probability distribution of magnetic hyperfine fields is shown on the right side of each spectrum. At a glance, Figure 3 shows the similar collapsing behavior of naked Fe_3_O_4_ NPs and NPs functionalized with CA (Fe_3_O_4_@CA and Fe_3_O_4_@CA@Dox). On the other hand, naked Fe_3_O_4_ NPs and NPs functionalized with LC (Fe_3_O_4_@LC and Fe_3_O_4_@LC@Dox) show a different collapsing behavior, which is also different among the three samples. This shows a clear difference in the magnetic structure of the two series of functionalized samples, which also gives a first hint for adding new magnetic spectral components in the MNPs functionalized with LC, as compared to the ones functionalized with CA. The evolution of the average magnetic hyperfine field, <B_hyp_>, versus temperature, as presented in Figure 4, can also be considered for a quantitative comparison of the two series of samples.

Notably, the almost similar thermal dependence of <B_hyp_>, together with the corresponding values of the average magnetic hyperfine field, for samples in the first series, namely naked Fe_3_O_4_, Fe_3_O_4_@CA, and Fe_3_O_4_@CA@Dox magnetic NPs, suggests similar values of anisotropy energy, KV (K is the anisotropy constant and V the MNP volume), for all NPs in the series. Assuming there is a similar average magnetic volume for all NPs in this series, the very similar magnetic relaxation, and hence KV products, leads to the conclusion of a very similar anisotropy constant in these samples. That is, similar magnetic configurations can be assumed for MNPs in this series, as it will be proven from the deep investigation of the low-temperature Mössbauer spectra presented in the next section.

Alternatively, the magnetic NPs in the second series (naked Fe_3_O_4_, Fe_3_O_4_@LC, and Fe_3_O_4_@LC@Dox) all show different and specific thermal behaviors, with enhanced magnetic relaxation (lower KV values) starting from the naked Fe_3_O_4_ and ending with Fe_3_O_4_@LC@Dox. Again, under the assumption of similar average size of nanoparticles in this second series, the direct influence of the surface molecules on the magnetic configuration of core MNPs can be directly deduced.

**Figure 5:**
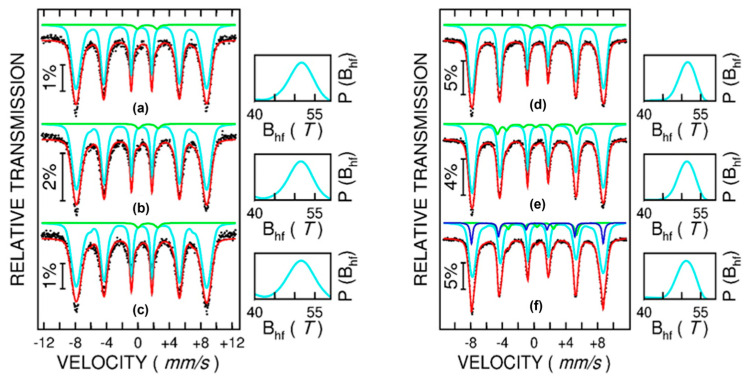
Mӧssbauer spectra collected at 6 K on naked and functionalized Fe_3_O_4_ NPs, as follows: naked Fe_3_O_4_ series 1 (**a**), Fe_3_O_4_@CA (**b**), Fe_3_O_4_@CA@Dox (**c**), naked Fe_3_O_4_ series 2 (**d**), Fe_3_O_4_@LC (**e**), and Fe_3_O_4_@CA@Dox (**f**). The probability distribution of the magnetic hyperfine field corresponding to the dominant broad sextet component (blue color) is shown on the right hand of each spectrum.

On the other hand, as evidenced by the 200 K Mӧssbauer spectra, the magnetic relaxation of the naked Fe_3_O_4_ NPs in the first series is slightly enhanced compared with the corresponding naked Fe_3_O_4_ NPs in the second series, under an almost identical magnetic hyperfine field value of 6 K and spontaneous magnetization. In these peculiar conditions, which suggest similar magnetic configurations of naked Fe_3_O_4_ NPs (e.g., similar magnetic configuration and K anisotropy constant) in the two series, the only explanation for the slightly faster relaxation of the naked MNPs in the first series is a slightly lower (e.g., by a few percent) average size of Fe_3_O_4_ NPs in this series, as compared to the Fe_3_O_4_ NPs in the second series. This aspect is not entirely unexpected given the criticality of NP size distribution on the processing conditions. However, such small differences in the size distribution of naked Fe_3_O_4_ NPs in the two series cannot be possibly evidenced by either TEM or SQUID investigations but only via the magnetic relaxation behavior within very short time windows, which is specific to Mӧssbauer spectroscopy. In the context of the high sensitivity of the Mӧssbauer spectra on the magnetic relaxation phenomena, the most proper investigation concerning the differences between the magnetic configuration of MNPs should be carried out at the lowest temperature of 6 K, where the MNPs are in a complete magnetically frozen regime (see Figure 5).

Concerning the Fe oxide MNPs in the first series, all spectra (Figure 5a–c) have been fitted by a highly dominant broad sextet component (over 98% relative area contribution), which is specific to distributed Fe configurations in a defect-like spinel structure of magnetite/maghemite type. The corresponding average isomer shift is similar in the case of all three samples in the series (naked Fe_3_O_4_, Fe_3_O_4_@CA, and Fe_3_O_4_@CA@Dox), i.e., 0.45(1) mm/s. This is the same with the average magnetic hyperfine field, i.e., 51.0(2) T, and the distribution width at half maximum, i.e., 6.4(2) T, which are the same for all samples in the series. These parameters point to MNPs of almost identical magnetic configurations in all three samples in the series, namely configurations based on Fe^3+^ ions in the high spin state of S = 5/2, i.e., with a defect spinel structure more closely resembling that of a maghemite. An almost insignificant central doubled component (less than 2% relative area contribution) was also considered for improving the fit quality in all spectra. This component can be associated with the specific hyperfine parameters (quadrupole splitting of 2.4(1) mm/s and isomer shift of 1.00 mm/s) to approximately less than 2% Fe^2+^ ions, most probable in very fine Fe oxide clusters, which are already superparamagnetic at 6 K. A negligible contribution to the magnetic state of the sample is expected from this less than 2% phase, which is however the same in all three samples. In this context, the almost identical Mӧssbauer spectra of the three samples in the series clearly prove that covering molecules does not change the magnetic configuration at the nanoparticle surface. While the Fe ions are the only magnetic ions, the conclusion is that CA molecules stick to the surface of MNPs, either by physical adsorption or by chemical bonds to the oxygen ions in the Fe oxide NPs. However, independent of the sticking mechanism, the conclusion of the Mössbauer investigation of the first series of samples is that the covering molecules do not affect the magnetic state of the core NPs. Therefore, the spontaneous magnetization of the magnetic core is the same for uncoated and surfacted MNPs, i.e., identical with the spontaneous magnetization of naked Fe_3_O_4_ (see Table 1). Hence, any variation in the spontaneous magnetization of the samples consisting of covered MNPs is strictly due to the amount of organic layers present in the sample. If M*_S_ is the spontaneous magnetization of the magnetic component (equal in this case to the spontaneous magnetization of naked Fe_3_O_4_ NPs) and M_S_ is the spontaneous magnetization of the covered MNPs, the amount of surface molecules (in grams) is straightforwardly obtained as

m_surf_ = 1 − M*_S_/M_S_ (1)

The loading is defined, in the present case, as the number of organic molecules expressed in g, which exists in 1 g of functionalized MNPs, namely m_surf_. The amount of magnetic material in 1 g of sample (functionalized MNPs) is evidently 1−m_surf_. Using the values of spontaneous magnetization obtained via the magnetic measurements, the following loadings were derived in the case of Fe_3_O_4_@CA and Fe_3_O_4_@CA@Dox MNPs: 0.055 g of CA molecules in 1 g of Fe_3_O_4_@CA sample and 0.165 g of CA@Dox molecules in 1 g of Fe_3_O_4_@CA@Dox sample. That is, an impressive loading by about 0.110 g of Dox (effective medicine molecule) has to be mentioned per g of Fe_3_O_4_@CA@Dox sample.

Notably, in reference [32], we have reported both the loading efficiency and the loading capacity with Dox molecules for the same Fe_3_O_4_@CA@Dox system by analyzing the amount of residual drug in the solution using spectrophotometry at 480 nm. Defining the loading capacity as the ratio between the amount of released drug and the total amount of nanoparticles (expressed as a percentage), a maximum value of 10(1)% was obtained for this system (prepared with 80 ppm Dox concentration), which is in very good agreement with the value reported in the present paper, reporting 0.11 g of Dox per g of coated Fe_3_O_4_@CA@Dox NPs.

Concerning the Fe oxide MNPs in the second series, the 6 K spectrum of naked Fe_3_O_4_ MNPs is very similar to the case of naked Fe_3_O_4_ MNPs in the first series (see Figure 5a,d) and has, therefore, been fitted accordingly. A first clear difference appears in the case of the 6 K spectrum of sample Fe_3_O_4_@LC (see Figure 5e) where an asymmetrically split sextet (7(1) % relative spectral area) has to be added to the main broad sextet (93(1) % relative spectral area) for a proper fit. Note that the hyperfine parameters of the main broad sextet are very similar to the ones for the naked Fe_3_O_4_ MNPs, pointing to the presence of Fe^3+^ ions in the high spin state S = 5/2. The hyperfine parameters assigned to the additional sextet are as follows: an isomer shift of 0.12(3) mm/s, a quadrupole splitting of 0.90(3) mm/s, and a magnetic hyperfine field of 31.0(1) T. These values are specific to Fe^3+^ ions in the intermediate spin state S = 3/2 [35] and lead to the conclusion that the LC coating molecules affect the magnetic state of the MNPs, most probably by changing (via chemical bonding) the local configuration of the Fe ions. For example, a distorted octahedral configuration (e.g., via a rhombic field) can lead to the proper splitting of the e_g_ and t_g_ orbitals, inducing an intermediate spin state. Moving on to the 6 K spectrum of sample Fe_3_O_4_@LC@Dox (see Figure 5f), an additional narrow sextet (13(1) % relative spectral area) should be added to the previously discussed spectral components (81(1) % spectral area of the main broad sextet and 6(1) % spectral area of the asymmetrically split sextet). These last two components keep closely their above mentioned hyperfine parameters. The hyperfine parameters corresponding to the additional component are the following: isomer shift of 0.35(1) mm/s, quadrupole splitting of 0.10(1) mm/s, and a magnetic hyperfine field of 51.5(1) T. These parameters indicate that this additional spectral component can be attributed to Fe^3+^ ions in a high spin state with S = 5/2; however, with a slightly different electron surrounding as compared to the Fe^3+^ ions evidenced in the initial naked Fe_3_O_4_ MNPs. In this respect, this third sextet component with much narrower spectral lines can be easily assigned to Fe^3+^ positions in very similar configurations as well-crystallized maghemite. That is, covering Dox molecules also slightly influence the magnetic state of NPs, in addition to the stronger influence coming from the CA covering molecules (some 87% of total Fe positions are similar to the case of Fe_3_O_4_@LC NPs)), giving rise to additional positions (some 13% of total Fe positions), which resemble those of hematite. Given this covering molecule effect, it becomes essential to estimate the real magnetic state of the covered MNPs, and hence the corresponding magnetization of the magnetic core, before applying relation (1) for deducing the weight of the surface molecules. This will be carried out in the following section through a proper corroboration of the Mössbauer and magnetic investigations.

It is worth mentioning here on the magnetic configuration of magnetite and the specific changes induced by the cation vacancies when moving toward the maghemite structure [37,38]. In a simplistic way, the unit formula of magnetite as an inverse spinel can be written as [Fe^3+^]_T_[Fe^3+^Fe^2+^]_Oh_O_4_, with one Fe^3+^ ion (S = 5/2) with a tetrahedral (T) coordination, one Fe^3+^ ion (S = 5/2) with an octahedral coordination, and one Fe^2+^ ion (S = 2) with an octahedral (Oh) coordination. Given the ferromagnetic coupling of spins in the same atomic site type and the antiferromagnetic coupling of spins in different types of atomic sites, a total spin of S = 2 can be assigned to the formula unit (f.u.) due to the compensation of the two spins belonging to Fe^3+^ ions on tetrahedral and octahedral positions. Hence, a magnetic moment of 4 µ_B_ is associated with the f.u. of well-crystallized magnetite and, finally, a spontaneous magnetization of 98 emu/g, as also reported in [39]. On the other hand, maghemite can be seen as a defected magnetite structure with cation vacancies on octahedral positions and only Fe^3+^ ions. Therefore, the f.u. can be simplified according to the following expression: [Fe^3+^]_T_[Fe^3+^Fe_x_^3+^]_Oh_O_4_-γ, with x = 0.66 and γ = 1. According to the above-mentioned spin coupling mechanisms, the corresponding magnetic moment per f.u. is 3.33 µ_B_, to which a spontaneous magnetization of 82 emu/g can be associated (again, similar to the maximum magnetization reported in [38]). Starting from both cases of magnetite and maghemite magnetic structures, a magnetization of about 24.7 emu/g can be associated with each Bohr magneton. Under the above assumptions, the spontaneous magnetization of only 62.9 emu/g associated with the naked MNPs, where only Fe^3+^ ions (more than 98%) have been evidenced by Mössbauer spectroscopy, should infer a maghemite-like structure with a value of x lower than 0.66. A magnetic moment per f.u. of only 2.54 µ_B_ corresponds to a spontaneous magnetization of 62.9 emu/g, following the above assumption of 24.7 emu/g associated with each Bohr magneton. Accordingly, the value of x = 0.51 corresponds to all f.u., which are not affected by the coating molecules. On the other hand, the affinity of organic molecules is expected to increase at the most defective sites, which are the octahedrally coordinated ones. This aspect is in agreement with the above observation that Fe^3+^ ions with intermediate spin states are the six coordinated ones, e.g., in the former octahedral sites. In this context, only 7% of the total Fe ions are affected by the organic molecules (see the relative spectral area of the Mössbauer component in Figure 5e), belonging to Fe^3+^ ions with S = 3/2), which means 0.17 Fe ions from a total 2.51 Fe ions per f.u. These 0.17 ions are part of the initial 0.51 ions with octahedral coordination, which contribute to the total magnetic moment of 2.54 µ_B/_f.u. of the naked Fe_3_O_4_ MNPs. As a consequence, the magnetic moment per f.u. in the magnetic core of the surfacted NPs can be expressed as μ = 0.17 × 3 μ_B_ + 0.34 × 5 μ_B_ = 2.21 μ_B_, where a magnetic moment of 3 μ_B_ and, respectively, of 5 μ_B_ has been assigned to Fe^3+^ ions with S = 3/2 and S = 5/2. Accordingly, a spontaneous magnetization of the magnetic core, M*_S_ of 54.6 emu/g, can be estimated in the case of sample Fe_3_O_4_@LC (see Table 2). Consequently, a loading of 0.044 g of LC molecules at 1 g of Fe_3_O_4_@LC NPs is estimated by relation (1).

In the case of sample Fe_3_O_4_@LC@Dox NPs, a direct and simple estimation of the magnetic moment in the magnetic core can also be obtained from the Mössbauer investigations. These show that in addition to the Fe ions in specific positions of Fe_3_O_4_@LC (characterized by a magnetic moment of 2.21 μ_B/_f.u. and accounted by the broad and the asymmetrical sextets), new configurations approaching the ones in well-crystallized maghemite (characterized by a magnetic moment of 3.33 μ_B/_f.u. and accounted by the third narrow sextet) are induced by the presence of Dox molecules. Considering the relative contributions provided by Mössbauer spectroscopy, the overall magnetic moment per f.u. in the magnetic core of Fe_3_O_4_@LC@Dox can be written as follows: μ = 0.87 × 2.21 μ_B_ + 0.13 × 3.33 μ_B_ = 2.36 μ_B_. Accordingly, the spontaneous magnetization of the magnetic core, M*_S_ of 58.3 emu/g, can be estimated in the case of sample Fe_3_O_4_@LC@Dox (see Table 2). A loading of 0.055 g of LC@Dox molecules at 1 g of Fe_3_O_4_@LC@Dox NPs are estimated by relation (1). Simply, it can be estimated that loading with Dox molecules is only 0.011 g per g of Fe_3_O_4_@LC@Dox NPs, which is about one order of magnitude lower than in the case of Fe_3_O_4_@CA@Dox NPs.

In order to compare this result with the spectrofluorimetric results on the same sample presented in [30], we should mention that the release of Dox strongly depends on the pH value of the buffer solution, with a quasi-complete release taking more than 90 h (there is high imprecision with regard to release time due to the asymptotic decrease in the Dox concentration in time). However, assuming a quasi-linear decrease in the Dox concentration in the PBS buffer solution, which extrapolates the data in Figure 8 of [30], an average release concentration per hour of about 4 µM of Dox (M = 543 g) can be assumed, together with a release time of 90 h, finally leading to about 2 µg/mL of total Dox. With the reported concentration of the NP solution (200 µg/mL), one obtains about 10 µg of Dox at 1000 µg of NPs, or equivalently, about 0.010 g of Dox at 1 g of NPs, which is in reasonable agreement with the results provided by the proposed methodology.

## 3. Materials and Methods

### 3.1. Materials

Iron (II) and (III) chloride, ammonia solution, L-Cysteine and ethanol were purchased from Sigma-Aldrich (St. Louis, MO, USA). Doxorubicin was acquired from AvaChem Scientific (San Antonio, TX, USA). All chemicals were used without purification and all the aqueous solutions were prepared using deionized water produced with a TKA-GenPure Water Purification System (TKA Wasseraufbereitungssysteme GmbH, Niederelbert, Germany).

### 3.2. Nanoparticle Synthesis

Magnetite nanoparticles (Fe_3_O_4_) were prepared via coprecipitation and coated with L-Cysteine (LC) and citric acid (CA) molecules, following the procedure reported in our previous works [30,39]. We started with a FeCl_3_ and FeCl_2_ ·4H_2_O solution with a 2:1 molar ratio (120 mL), over which an ammonia solution (30 mL; 25%) was added dropwise. Subsequent functionalization with doxorubicin (resulting in Fe_3_O_4_@LC@Dox and Fe_3_O_4_@CA@Dox) was completed through continuous stirring for 24 h, according to the same works [30,39]. Two sets of uncoated Fe_3_O_4_ nanoparticle samples were prepared within similar processing conditions, as starting samples denoted in the following naked Fe_3_O_4_, in the two considered functionalization branches based on LC and CA, respectively.

### 3.3. Methods

The magnetic investigations regarding the relaxation phenomena were performed by acquiring Zero Field Cooled—Field Cooled (ZFC-FC) magnetization curves in a 100 Oe applied field. Hysteresis loops were recorded in magnetic fields of up to 2T induction fields (20,000 Oe intensity) at 10 K and 300 K for all samples (naked Fe_3_O_4_, Fe_3_O_4_@LC, Fe_3_O_4_@LC@Dox, Fe_3_O_4_@CA, and Fe_3_O_4_@CA@Dox MNPs). The measurements were carried out using a SQUID magnetometer (MPMS7, from Quantum Design, San Diego, CA, USA). In addition, ^57^Fe Mössbauer spectra have been collected at low temperatures from 6 K to room temperature (RT) by using a Mössbauer spectrometer with a linear waveform (SEECo, Minneaplolis, MN, USA) and a ^57^Co(Rh) radioactive source with a 50 mCi initial activity. In this respect, the samples were introduced in a close cycle Mössbauer cryostat (Janis, Woburn, MA, USA). The NORMOS software [40] was used for fitting the Mössbauer spectra via hyperfine magnetic field distributions, according to the algorithm specific to MNPs with defective magnetic structures [36]. The isomer shifts are reported relative to α-Fe at room temperature.

## 4. Conclusions

The proposed methodology for the sensitive evaluation of the loading of MNPs by organic molecules is exemplified in the case of two series of MNPs prepared in similar conditions as follows: (i) a first series consisting of naked Fe_3_O_4_, Fe_3_O_4_@CA, and Fe_3_O_4_@CA@Dox MNPs; and (ii) a second series consisting of naked Fe_3_O_4_, Fe_3_O_4_@LC, and Fe_3_O_4_@LC@Dox MNPs.

It has been shown that the LC molecules affect the magnetic configuration of the magnetic core (reflected by an additional spectral component in the low-temperature Mössbauer spectrum, as compared to the case of naked Fe_3_O_4_ NPs), as well as the bounding of subsequent Dox molecules (which provides an additional third component in the Mössbauer spectrum, as compared to the case of Fe_3_O_4_@LC NPs). The carefully complementary low-temperature magnetic and Mössbauer investigations show a loading of 0.044 g of LC molecules at 1 g of Fe_3_O_4_@LC NPs and of 0.055 g of LC@Dox molecules at 1 g of Fe_3_O_4_@LC@Dox NPs. The resulting loading with about 0.011 g of Dox at 1 g of Fe_3_O_4_@LC@Dox NPs is in reasonable agreement with the loading estimated from the time evolution of the released Dox concentration in PBS buffer solution, as presented in [30].

By contrast, the covering molecules of CA do not influence the magnetic state of the MNPs, which also remain unchanged by the subsequent addition of Dox molecules, according to the low-temperature Mössbauer investigations. Loading with CA molecules is 0.055 g for 1 g of Fe_3_O_4_@CA NPs (comparable with the case of loading LC molecules) and 0.165 g for CA@Dox molecules in 1 g of Fe_3_O_4_@CA@Dox NPs (much higher than in the case of Fe_3_O_4_@LC@Dox NPs), given that the surface of the magnetic core is still not affected. This aspect may suggest that the binding of the Dox molecules in the Fe_3_O_4_@CA@Dox NP system is realized through the CA surface molecules, which have a high affinity for Dox molecules. As a consequence, an order of magnitude higher loading by Dox medicine molecules is evidenced in Fe_3_O_4_@CA@Dox NPs (0.11 g per g coated MNPs), as compared to the Fe_3_O_4_@LC@Dox NPs (0.011 g per g coated MNPs). The loading by 0.11 g of Dox per g of coated Fe_3_O_4_@CA@Dox NPs agrees well with the loading capacity reported for the same system in [32].

## Figures and Tables

**Table 1:** Blocking temperature, spontaneous magnetization, corrected magnetization of the magnetic core, and loading in grams of surface molecules per gram of loaded magnetic material (CA branch). Numbers in brackets represent the errors of the last-mentioned digit.

Sample	Naked Fe_3_O_4_	Fe_3_O_4_@CA	Fe_3_O_4_@CA@Dox
T_B_ (K)	218(2)	220(2)	220(2)
M_S_ (emu/g)	62.8(3)	59.3(3)	52.4(3)
M*_S_ (emu/g)	-	62.8(3)	62.8(3)
Loading (g surf/g sample)	-	0.055(5)	0.165(5)

**Table 2:** Blocking temperature, spontaneous magnetization, spontaneous magnetization of the magnetic core, and loading in grams of surface molecules per gram of loaded magnetic material (LC branch). Numbers in brackets represent the errors of the last-mentioned digit.

Sample	Naked Fe_3_O_4_	Fe_3_O_4_@LC	Fe_3_O_4_@LC@Dox
T_B_ (K)	220(2)	218(2)	210(2)
M_S_ (emu/g)	62.9(3)	52.2(3)	55.1(3)
M*_S_ (emu/g)	-	54.6(3)	58.3(3)
Loading (g surf/g sample)	-	0.044(5)	0.055(5)

## Data Availability

Magnetic and Mössbauer data can be available at request.

## References

[1] McNamara K., Tofail S.A.M. (2017). Nanoparticles in Biomedical Applications. Adv. Phys. X.

[2] Diez-Pascual A.M., Rahdar A. (2022). Functional Nanomaterials in Biomedicine: Current Uses and Potential Applications. ChemMedChem.

[3] Ulbrich K., Hola K., Šubr V., Bakandritsos A., Tuček J., Zbořil R. (2016). Targeted Drug Delivery with Polymers and Magnetic Nanoparticles: Covalent and Noncovalent Approaches, Release Control, and Clinical Studies. Chem. Rev..

[4] Liu X., Zhang Y., Wang Y., Zhu W., Li G., Ma X., Zhang Y., Chen S., Tiwari S., Shi K. (2020). Comprehensive Understanding of Magnetic Hyperthermia for Improving Antitumor Therapeutic Efficacy. Theranostics.

[5] Wang M., Jin L., Leung P.H.-M., Chow F.W.-N., Zhao X., Chen H., Pan W., Liu H., Li S. (2024). Advancements in Magnetic Nanoparticle-Based Biosensors for Point-of-Care Testing. Front. Bioeng. Biotechnol..

[6] Han X., Xu K., Taratula O., Farsad K. (2019). Applications of Nanoparticles in Biomedical Imaging. Nanoscale.

[7] Brown J.S., Amend S.R., Austin R.H., Gatenby R.A., Hammarlund E.U., Pienta K.J. (2023). Updating the Definition of Cancer. Mol. Cancer Res..

[8] Boogaard W.M.C., Komninos D.S.J., Vermeij W.P. (2022). Chemotherapy Side-Effects: Not All DNA Damage Is Equal. Cancers.

[9] Dhara A., Majumder S., Pahari S., Kar D. (2023). Improved Targeting of Therapeutics by Nanocarrier-Based Delivery in Cancer Immunotherapy and Their Future Perspectives. BioNanoScience.

[10] Zhou X., He X., Dong Z., Wang Y., Hu C., Zhang D., Guo R., Qiao J., Li N. (2024). Manganese-Doped Mesoporous Magnetic Nanocarriers for Cancer Treatment. ACS Appl. Nano Mater..

[11] Ozpolat B., Sood A.K., Lopez-Berestein G. (2014). Liposomal siRNA Nanocarriers for Cancer Therapy. Adv. Drug Deliv. Rev..

[12] Zhang W., Taheri-Ledari R., Ganjali F., Mirmohammadi S.S., Qazi F.S., Saeidirad M., KashtiAray A., Zarei-Shokat S., Tian Y., Maleki A. (2023). Effects of Morphology and Size of Nanoscale Drug Carriers on Cellular Uptake and Internalization Process: A Review. RSC Adv..

[13] Donahue N.D., Acar H., Wilhelm S. (2019). Concepts of Nanoparticle Cellular Uptake, Intracellular Trafficking, and Kinetics in Nanomedicine. Adv. Drug Deliv. Rev..

[14] Liu X., Cheng Y., Mu Y., Zhang Z., Tian D., Liu Y., Hu X., Wen T. (2024). Diverse Drug Delivery Systems for the Enhancement of Cancer Immunotherapy: An Overview. Front. Immunol..

[15] Beach M.A., Nayanathara U., Gao Y., Zhang C., Xiong Y., Wang Y., Such G.K. (2024). Polymeric Nanoparticles for Drug Delivery. Chem. Rev..

[16] Li J., Wang Q., Xia G., Adilijiang N., Li Y., Hou Z., Fan Z., Li J. (2023). Recent Advances in Targeted Drug Delivery Strategy for Enhancing Oncotherapy. Pharmaceutics.

[17] Du S., Guan Y., Xie A., Yan Z., Gao S., Li W., Rao L., Chen X., Chen T. (2023). Extracellular Vesicles: A Rising Star for Therapeutics and Drug Delivery. J. Nanobiotechnol..

[18] Attia M.F., Anton N., Wallyn J., Omran Z., Vandamme T.F. (2019). An Overview of Active and Passive Targeting Strategies to Improve the Nanocarriers Efficiency to Tumor Sites. J. Pharm. Pharmacol..

[19] Bertrand N., Wu J., Xu X., Kamaly N., Farokhzad O.C. (2014). Cancer Nanotechnology: The Impact of Passive and Active Targeting in the Era of Modern Cancer Biology. Adv. Drug Deliv. Rev..

[20] Narum S.M., Le T., Le D.P., Lee J.C., Donahue N.D., Yang W., Wilhelm S. (2020). Passive Targeting in Nanomedicine: Fundamental Concepts, Body Interactions, and Clinical Potential. Nanoparticles for Biomedical Applications.

[21] Sritharan S., Sivalingam N. (2021). A Comprehensive Review on Time-Tested Anticancer Drug Doxorubicin. Life Sci..

[22] Lee J., Choi M.-K., Song I.-S. (2023). Recent Advances in Doxorubicin Formulation to Enhance Pharmacokinetics and Tumor Targeting. Pharmaceuticals.

[23] Mattioli R., Ilari A., Colotti B., Mosca L., Fazi F., Colotti G. (2023). Doxorubicin and Other Anthracyclines in Cancers: Activity, Chemoresistance and Its Overcoming. Mol. Asp. Med..

[24] Yang F., Teves S.S., Kemp C.J., Henikoff S. (2014). Doxorubicin, DNA Torsion, and Chromatin Dynamics. Biochim. Biophys. Acta Rev. Cancer.

[25] Kciuk M., Gielecińska A., Mujwar S., Kołat D., Kałuzińska-Kołat Ż., Celik I., Kontek R. (2023). Doxorubicin—An Agent with Multiple Mechanisms of Anticancer Activity. Cells.

[26] Belger C., Abrahams C., Imamdin A., Lecour S. (2024). Doxorubicin-Induced Cardiotoxicity and Risk Factors. IJC Heart Vasc..

[27] Rosenblatt K.M., Bunjes H. (2017). Evaluation of the Drug Loading Capacity of Different Lipid Nanoparticle Dispersions by Passive Drug Loading. Eur. J. Pharm. Biopharm..

[28] Zhang L., Zhu H., Gu Y., Wang X., Wu P. (2019). Dual Drug-Loaded PLA Nanoparticles Bypassing Drug Resistance for Improved Leukemia Therapy. J. Nanopart. Res..

[29] Wilkosz N., Łazarski G., Kovacik L., Gargas P., Nowakowska M., Jamroź D., Kepczynski M. (2018). Molecular Insight into Drug-Loading Capacity of PEG−PLGA Nanoparticles for Itraconazole. J. Phys. Chem. B.

[30] Toderascu L.I., Sima L.E., Orobeti S., Florian P.E., Icriverzi M., Maraloiu V.-A., Comanescu C., Iacob N., Kuncser V., Antohe I. (2023). Synthesis and Anti-Melanoma Activity of L-Cysteine-Coated Iron Oxide Nanoparticles Loaded with Doxorubicin. Nanomaterials.

[31] Ural M.S., Dartois E., Mathurin J., Desmaële D., Collery P., Dazzi A., Deniset-Besseau A., Gref R. (2022). Quantification of Drug Loading in Polymeric Nanoparticles Using AFM-IR Technique: A Novel Method to Map and Evaluate Drug Distribution in Drug Nanocarriers. Analyst.

[32] Sima L., Toderascu L.I., Tudor M., Florian P., Icriverzi M., Ionita F., Maraloiu V., Iacob N., Kuncser V., Antohe I. (2024). In Vitro and In Vivo Investigations of Citric Acid Functionalized Magnetic Iron Oxide Nanoparticles for Intra-Tumoral Melanoma treatment. Nanoscale Adv..

[33] Craciunescu I., Palade P., Iacob N., Ispas G.M., Stanciu A.E., Kuncser V., Turcu R.P. (2021). High-Performance Functionalized Magnetic Nanoparticles with Tailored Sizes and Shapes for Localized Hyperthermia Applications. J. Phys. Chem. C.

[34] Schinteie G., Kuncser V., Palade P., Dumitrache F., Alexandrescu R., Morjan I., Filoti G. (2013). Magnetic Properties of Iron–Carbon Nanocomposites Obtained by Laser Pyrolysis in Specific Configurations. J. Alloys Compd..

[35] Greenwood N.N., Gib T.C. (1971). Mossbauer Spectroscopy.

[36] Kuncser V., Schinteie G., Sahoo B., Keune W., Bica D., Vekas L., Filoti G. (2007). Magnetic Interactions in Water Based Ferrofluids Studied by Mossbauer Spectroscopy. J. Phys. Condens. Matter..

[37] Kuncser V., Palade P., Kuncser A., Greculeasa S., Schinteie G. (2014). Engineering Magnetic Properties of Nanostructures via Size Effects and Interphase Interactions. Size Effects in Nanostructures: Basics and Applications.

[38] Kuncser V., Crisan O., Schinteie G., Tolea F., Palade P., Valeanu M., Filoti G. (2013). Magnetic Nanophases: From Exchange Coupled Multilayers to Nanopowders and Nanocomposites.

[39] Nguyen M.D., Tran H.-V., Xu S., Lee T.R. (2021). Fe_3_O_4_ Nanoparticles: Structures, Synthesis, Magnetic Properties, Surface Functionalization, and Emerging Applications. Appl. Sci..

[40] Brand R.A. (1987). Improving the Validity of Hyperfine Field Distributions from Magnetic Alloys: Part I: Unpolarized Source. Nucl. Instrum. Methods Phys. Res. Sect. B Beam Interact. Mater. At..

